# Risk factors of ocular morbidity among under‐five years old children in Khartoum State‐ Sudan‐ 2020

**DOI:** 10.1002/hsr2.279

**Published:** 2021-05-03

**Authors:** Mohanad Kamaleldin Mahmoud Ibrahim, Jacqueline Elizabeth Wolvaardt, Mustafa Khidir Mustafa Elnimeiri

**Affiliations:** ^1^ Community Medicine & Epidemiology, Faculty of Medicine, Department of Community Medicine Ibn Sina University Khartoum Sudan; ^2^ School of Health Systems and Public Health University of Pretoria South Africa; ^3^ Preventive Medicine & Epidemiology Alneelain University Khartoum Sudan

**Keywords:** epidemiology, opthalmology, pediatrics, public health

## Abstract

**Background and Aims:**

Visual impairment in early childhood can significantly affect the development of visual, motor, and cognitive function and potentially lead to long‐term adverse psychosocial consequences. This study aimed to identify the risk factors of ocular morbidity among under 5‐year old children in Khartoum State, Sudan.

**Methods:**

A cross‐sectional study was conducted in three tertiary eye care hospitals in Khartoum State, Sudan. The study included 391 children under the age of 5 years. The parent(s) were interviewed using a precoded, pretested, closed‐ended questionnaire that included questions regarding socio‐demographic profile and possible risk factors. Data were analyzed using Statistical Package for the Social Sciences (version 21.0). A *P*‐value of less than .05 was regarded as significant.

**Results:**

There was a significant association between participants with diabetes mellitus and poor vision (*P*‐value <.001). Two‐thirds of participants (57%) with visual impairment had mothers, who reportedly attended antenatal care services regularly (*P*‐value .001), revealing a significant statistical association. Maternal diseases, specifically diabetes, was identified as a risk factor for poor visual acuity in their offspring (*P*‐value <.001). A significant relation was revealed between family history of eye disease and the degree of relationship to the affected participant (*P*‐value <.001). There was an association between watching TV and current visual acuity (*P*‐value <.001); as well as using mobile phones and current visual acuity (*P*‐value <.001). Multilinear analysis revealed the stronger influence of TV watching rather than the use of mobile phones (*P*‐value <.001).

**Conclusions:**

Diabetes, diabetic mothers, a family history of ocular morbidity, watching television, and using mobile phones emerged as significant risk factors of ocular morbidity among children under the age of 5 years in this study. Many of these risk factors are either modifiable or controllable ocular morbidities among under‐five children can be reduced with suitable interventions.

**Abbreviations**
VI: vision impairment
SVI: severe visual impairment
ROP: retinopathy of prematurity
SPSS: Statistical Package for the Social Sciences (SPSS)
DM: diabetes mellitus
TV: television
OLSM: The Orinda Longitudinal Study of Myopia
HMW: high‐molecular‐weight
PCG: primary congenital glaucoma
IOP: intraocular pressure
RGC: retinal ganglion cells
DED: dry eye disease
QoL: quality of life
RF‐EMFs: radiofrequency‐modulated electromagnetic fields (RF‐EMFs)

## 1 BACKGROUND

Children are vital to nations' present and future. Measurement and appropriate use of data on children's health and social determinants of health can help ensure that policies are based on good information and are designed to enhance the health of children.^1^

Most eye diseases affect the vision. Vision impairment (VI) is a disability that represents a cost to the individual, society, and the health budget.

The development of child's visual system starts intrauterine and continues after birth with progressive development over the early few months after birth. At age of 2 months, the child will be able to establish hand‐eye coordination. Children will be able to judge an object distance (depth perception) at around the age of 5 months.^2^ Vision accommodation and eyes movement in opposite direction develop between the ages of 3 and 5 months.^3^

Visual impairment has negative impacts on the development of the children that include visor, motor, and cognitive defects. Multiple studies reported untreated refractive errors as a predisposing factor of poor literacy in under‐5 year old children while correction of refractive errors in preschooler can bring back their visual motor function.^4^, ^5^, ^6^, ^7^, ^8^

In high‐income countries, the leading causes of severe visual impairment (SVI) among children are related to cerebral disorders and optic nerve anomalies. Other common ocular diseases are retinopathy of prematurity (ROP) and cataract. Nowadays, in low‐ income countries, the causes of SVI are almost similar to the those in high‐income countries where they used to be related to infectious, nutrional eye diseases, and congenital anomalies in the past. This shift in the pattern of ocular diseases is directly related to the improvement in maternal and neonatal healthcare and to the strengthening of eye healthcare services.^9^

It is highly predicted that, the prevalence of VI among preschoolers will increase. Thus, early detection of ocular disorder and preventive measures are necessary in order to improve the under‐5 year old health and preserve their natural development.^10^ As the majority of ocular diseases are either preventable or treatable,^11^ increasing the level of awareness toward eye health will incorporate in improving the community eye health.

The ultimate goal of this study is to identify the risk factors of ocular morbidity among under 5‐year old children in Khartoum State, Sudan.

## 2 METHODS

A cross‐sectional study was conducted in three busy tertiary eye care hospitals in Khartoum State. The sum of the three hospitals' outpatient numbers is approximately 40 000 per month, which include 1500 children. The combined monthly surgeries are approximately 5500, which include approximately 200 surgeries performed on children. These hospitals each have pediatrics ophthalmology departments with a combined number of nine pediatric consultant ophthalmologists. These hospitals receive pediatric patients from the geographical area of Khartoum State as well as children referred from other states. The selected hospitals are considered the largest in the state with an independent pediatric ophthalmology unit and specialized doctors in pediatrics ophthalmology. They serve the majority of primary and referred children with ocular disorders.

### 2.1 Sample size and sampling process

The sample size was estimated using the population formula where the population was the total number of pediatric patients attended in the last year across the three hospitals. N/1 + N (d ^2^).

N = 17 314/1 + 17 314(0.05 * 0.05) = 391.

Cluster sampling was used to draw the sample proportionate to the size of the study population at each hospital, where each hospital was considered as a cluster and then the number of participants was estimated proportionally according to hospital number of pediatric patients in the past 1 year. Convenience sampling was performed to select the participants, who fulfilled the inclusion criteria. These criteria included, being a boy or girl under 6 years of age, attending pediatric ophthalmology clinic to the first time, and his/her parent (s) voluntary agreed to participate in the study. All children of more than 5 years old were excluded.

Data were collected via interviews conducted onsite with one or more of the parents of the participants. The parent(s) were interviewed using a precoded, pretested, and closed‐ended questionnaire that included questions related to socio‐demographic data and possible risk factors identified in the literature. The questionnaire was pretested using pilot survey and modified accordingly, with insuring to include these participants in the study to avoid bias. Participants' clinical information was obtained from the consultant who performed the clinical examinations. We considered good vision as any visual acuity not worse than 6/12, poor vision as any visual acuity better than 6/12 and up to 3/60, and blindness as presenting visual acuity of less than 3/60 in the better eye.

### 2.2 Data management and analysis

The data were sorted, cleaned, categorized, and summarized on a master sheet and then analyzed using Statistical Package for the Social Sciences (SPSS) version 21.0. The analysis encompasses descriptive univariate information, bi‐variate analysis for cross‐tabulation using chi‐square test for associations, and multilinear regression analysis for differences among the variables of interest. A *P*‐value of less than .05 was regarded as significant.

### 2.3 Ethics and permissions

The study was reviewed and approved by Albasar Institutional Review Board (B‐IRB‐20‐MR‐012). Permission to conduct the research was obtained from each hospital and informed written consent was obtained from the children's parent for both participation in the study and publication of the findings.

In this study, the anonymity of hospitals and patients was assured as only aggregated data is presented. All necessary measures were taken to ensure the confidentiality of the collected information. During the data collection, the records were never left unattended and they were stored in a locked room when not in use. Data were directly entered into a password protected master sheet.

All authors declare no conflict of interest and this study was not funded.

## 3 RESULTS

The majority of the interviewees were the children's mothers (71%), a small majority (54%) of whom were housewives. Approximately a quarter (23%) of interviewees were fathers, where less than half (38%) were working at professional jobs and the remaining fathers were evenly spread among private, governmental, and physical labor jobs. The remaining (6%) were participants' relatives. Most of the participants' families could be considered as stable as only 15% were either divorced or separated. In this group, 18% of participating children had one parent who died (65% were fathers and 35% were mothers).The mean income of the participants' families was 15 278 Sudanese pounds per month and the mean number of family members was 4 (Table 1).

**TABLE 1:** Background characteristics of study participants

Characteristic			Percentage
Interviewee	Mothers		71%
	Fathers		23%
	Other relatives		6%
Occupation of parents	Mothers	Housewives	54%
		Professional	15%
		Private	15%
		Governmental	6%
		Others	10%
	Fathers	Unemployed	12%
		Professional	38%
		Private	18%
		Governmental	16%
		Others	16%
Deceased parent (s)	Percentage of participants with a deceased parent		18%
Marital status of parents	Married		85%
	Divorced/Separated		15%
Number of family members	Mean number of family members		4
Family income	Monthly mean families income		15 278 SDG

There was an association between diabetes mellitus (DM) and poor vision (*P*‐value <.001) revealed by cross‐tabulation test using Chi‐square test (Table 2). Almost two‐thirds (57%) of children with visual impairments had mothers, who reported regular attendance of antenatal care services, revealing a significant statistical association (*P*‐value .001; Table 3). Mothers who reported having a disease during pregnancy exemplify a significant risk factor relating to eye morbidity, especially vision impairment in their offspring (*P*‐value <.001).Among these diseases, DM was the most prevalent (Table 4). There was a significant association between the family history of eye diseases and the degree of relationship of the affected family member (*P*‐value <.001; Table 5). The closer the relationship of the affected family member to the participant, the higher the risk of developing an eye disorder for that child. Refractive errors were the most common reported condition among family members.

**TABLE 2:** Association between current vision and children's chronic conditions

Current vision	Hypertension	Diabetes	No	Total
Good	12	0	112	124
Poor	0	11	224	235
Blind	0	0	32	32
Total	12	11	368	391

*Note: P*‐value = <.001.

**TABLE 3:** Association between current vision and reported attendance of antenatal care services

Current vision	Regular antenatal care		Total
	Yes	No	
Good	89	35	124
Poor	190	45	235
Blind	32	0	32
Total	311	80	391

*Note: P*‐value = .001.

**TABLE 4:** Association between current vision with maternal disease during pregnancy

Current vision	Maternal disease during pregnancy			Total
	Diabetes	Hypertension	Eclampsia	
Good	22	21	0	43
Poor	46	13	26	85
Blind	11	0	0	11
Total	79	34	26	139

*Note: P*‐value <.001.

**TABLE 5:** Association between a family history of eye disease and the degree of relationship for children with ocular morbidity (N = 391)

Type of disease	Degree of relationship			Total
	First	Second	Third	
Refractive errors	38	0	0	38
Cataract	17	0	0	17
Glaucoma	9	5	0	14
Retina	3	0	0	3
No disease	21	6	12	39
Total	88	11	12	111

*Note: P*‐value <.001.

Nearly 40% of the study participants reportedly watched television (TV); (20%) occasionally, and (19%) more than 2 hours per day. Watching TV was strongly associated with current vision status (*P*‐value <.001; Table 6). More than one‐third (33%) of study participants reportedly used mobile phones. There was a significant association between the duration of mobile phone usage and poor vision, the longer duration of mobile phone usage associated with the frequency of participants with poor visual acuity (*P*‐values <.001; Table 7). A multilinear regression analysis was performed to examine the relationship between TV watching and mobile use and visual acuity. This analysis revealed a significant difference in favor of TV watching making it a stronger associated risk factor for visual acuity (*P*‐value <.001) in this study (Table 8).

**TABLE 6:** Association between current vision and reported duration of watching TV

Current vision	Duration of watching TV			Total
	Occasionally	>2 hours per day	No	
Good	10	11	103	124
Poor	56	51	128	235
Blind	11	11	10	32
Total	77	73	241	391

*Note: P*‐value <.001.

**TABLE 7:** Association between current vision and use of a mobile phone

Current vision	Duration of using mobile phones				Total
	Occasionally	<2 hours daily	>2 hours daily	No	
Good	10	11	0	103	124
Poor	34	22	40	139	235
Blind	0	0	11	21	32
Total	44	33	51	263	391

*Note: P*‐value <.001.

**TABLE 8:** Current vision with daily TV and daily use of mobile phone

Model	Unstandardized coefficients		Standardized coefficients	*t*	Sig.
	*B*	Std. error	Beta		
(Constant)	1.978	0.116		17.105	.000
Daily TV	−0.238	0.035	−0.472	−6.802	.000
Daily use of mobile	0.138	0.044	0.244	3.124	.002

*Note: P*‐value <.001. *Dependent Variable*: Current vision status. *Predictors*: (Constant), Watch TV, Daily use of mobile phones on mobile.

## 4 DISCUSSION

This study was conducted to identify the common risk factors associated with ocular morbidity among under‐five children aiming to identify possible missed opportunities for prevention and control.

There were marginally more male participants in this study (male to female ratio was 1.1 to 1).These socio‐demographic background of the interviewees is representative of Sudanese life in general.^12^, ^13^

The association between participants with DM and poor vision was expected as DM is a disease that affects the micro‐capillaries of the body system and the retina is part of the affected tissues. Bjornstad reported in his study that a major complication of DM in childhood is retinal and corneal changes that in turn affects vision due to early involvement of the micro‐vessels.^14^

Large disparities exist in perinatal health, not only between countries but also within cities and population groups.^15^ The study yielded an unexpected association between the majority of participants with visual impairment and reported regular attendance of antenatal care services. Clearly, attendance of antenatal care services cannot be a risk factor so this finding is likely to be coincidental. The majority of early neonatal disorders are related to medically related factors that can be prevented and managed with the access to antenatal care services. The remaining problems of nonmedical risks such as social, mental, and barriers of access to proper care are factors that significantly hazardous to maternal and child health.^16^

In this study, participants with poor visual acuity were significantly more likely to have mothers with DM. This finding is similar to other studies that link children's vision to maternal health status. A study by Borchertet al. report that children whose mothers had a history of maternal smoking during pregnancy were more likely (OR, 1.4) to have hyperopia and cessation of maternal smoking during pregnancy may reduce the risk of hyperopia in these children.^17^ The literature documented a relation between maternal DM and the child health including vision. Intrauterine metabolic experiences continue to influence the neuro‐developmental course in offspring of mothers with diabetes. Diabetes management and obstetric and neonatal care appear to effectively mitigate the potential long‐term effects of most perinatal complications and morbidities. One study conducted by Rizzo revealed a borderline association between the children's scores on the psychomotor development index at 2 years of age and maternal third‐trimester β‐hydroxybutyrate levels.^18^ Congenital hypoplasia of the optic nerve is consequent to maternal DM and it should be included in the differential diagnosis of visual field defects even when they are discovered in adults.^19^

This study confirmed the association between a close member of the family, who has an eye disorder, and the visual acuity of participants. Refractive errors affecting first‐degree relatives were the predominant type of eye disorders. There is strong evidence from the literature that, myopic parents are more prone to have myopic children.^20^ The Orinda Longitudinal Study of Myopia (OLSM) revealed a significant association between parental myopia and the development of refractive error and axial length followed by myopia among their children.^21^ Multiple studies have documented a higher prevalence of myopic children among families where both parents have myopia, while lower prevalence was noticed in families where no or only one parent is affected.^22^, ^23^, ^24^, ^25^, ^26^, ^27^, ^28^

Ocular morbidity was associated with a family history of cataracts among first‐degree relatives.^29^ Juvenile cataract is a leading cause of VI in children. Cataract is a disorder that interrupts the passage of light due to disruption in the consistency of the lens. In young children, the pathophysiology is related to high‐molecular‐weight (HMW) protein aggregates and the some inherited genes, while in older ages, it is related to some environmental factors. The best visual outcome can be achieved with early surgical intervention in the first 6 weeks of age.^30^ Despite the reduction in prevalence of congenital rubella, the percentage of congenital cataract caused by unidentified factors, genetic and infectious diseases remains high. These factors make the prevention of congenital cataract challenging.^31^

In this study, glaucoma among children was associated with a family history of glaucoma among first‐degree relatives. Glaucoma is a condition that can be inherited as a Mendelian autosomal dominant/recessive trait, or as a complex trait. Different genes have been discovered with multiple interacting loci.^32^

Glaucoma is the largest cause of irreversible blindness and 6.9 million people globally are visually impaired due to glaucoma.^33^, ^34^ Primary congenital glaucoma (PCG) is a rare disease affecting children early in life. PCG was considered untreatable with inevitable blindness. Family history is one of the major risk factors for this disease, and it comprises a heterogeneous group of conditions that damage retinal ganglion cells (RGC) and the optic nerve.^35^, ^36^

Watching TV for a long duration at this age is a significant risk to developing eye disorder(s). Nearly 40% of the study participants watch TV occasionally or for more than 2 hours per day. Watching TV showed significant association with current vision status. In addition, the multilinear regression analysis revealed that TV watching, more than use of a mobile phone, was the strongest factor linked to visual acuity in this sample. The study by Bener and et al. reported that low vision was more prevalent among frequent television viewers (17.2%) than in infrequent viewers (14.0%). The study also confirmed that the proportion of children who wear glasses was higher among those who were frequent TV viewers (21.3%).^37^ In one Ethiopian study, sex, age, school type, television exposure duration, the distance of television exposure, mobile phone exposure, and medical visits were factors associated with visual impairment. Raising the level of awareness across community individuals toward early detection of VI and providing affordable eye healthcare services might reduce its prevalence.^38^

Using mobile phones was another factor associated with an increased risk on visual acuity in this sample. Although 67% of study participants reportedly did not use mobile phones, there was a significant association between using mobile phone and poor vision. This association was more significant with those exposed to mobile phones for more than 2 hours per day. These findings confirm what was previously reported where smartphone use in children was strongly associated with pediatric dry eye disease (DED); whereas the prevalence of DED is related to the longer mean daily duration of smartphone use.^39^, ^40^ DED is public health concern that results in a multifaceted degradation of patients' quality of life (QoL) and visual performance. It has a measureable impact on several aspects of patients' QoL including pain, vitality, and ability to perform certain activities requiring sustained visual attention.^41^ Exposure to radiofrequency‐modulated electromagnetic fields (RF‐EMFs) produced by cell phones has possible harmful consequences including carcinogenic effects. These effects provoked worries regarding the usage of cell phones.^42^

According to the literature, children are more sensitive to radiation than adults.^43^ Other studies have reported that the bone marrow of children absorbs 10 times more microwave radiation than adults. Germany and other technologically advanced countries have approved laws issue warnings about youngsters' use of wireless devices.^44^

## 5 CONCLUSIONS

This study confirms that children under the age of 5 years who have DM, or whose mothers had DM, or who have a family history of ocular morbidity, or who watch TV daily or use mobile phones for more than 2 hours per day are all associated with ocular morbidities that negatively affect the visual acuity. Thus, ocular morbidities among under‐five children can be reduced if suitable intervention toward prevention of modifiable risk factors and control of the nonmodifiable ones has been made.

## 6 LIMITATIONS OF THE STUDY

This study is limited to small geographical area of the country. The results of this study cannot be extrapolated to the entire country, but they can be representative for the capital state. This study is expected to be useful for exploring the commonest related risks of ocular diseases among under‐5 years children and therefore helpful for prevention and control planning by the decision makers and professionals in the field.

The sample size was relatively small as the design effect was not considered due to resources limitations, but study is believed to be valid according to its aim where its planned to highlight on a problem among small proportion of the community concerning a relatively non frequent disorders.

## FUNDING

None.

## CONFLICT OF INTEREST

The authors declare they have no conflict of interest.

## AUTHOR CONTRIBUTIONS

Conceptualization: Mohanad Ibrahim

Formal Analysis: Mohanad Ibrahim

Writing ‐original draft: Mohanad Ibrahim

Writing ‐ review and editing: Jacqueline Wolvaardt, Mustafa Elnimeiri, Mohanad Ibrahim

All authors have read and approved the final version of the manuscript.

Mohanad Ibrahim had full access to all of the data in this study and takes complete responsibility for the integrity of the data and the accuracy of the data analysis.

## TRANSPARENCY STATEMENT

Mohanad Ibrahim affirms that this manuscript is an honest, accurate, and transparent account of the study being reported; that no important aspects of the study have been omitted; and that any discrepancies from the study as planned (and, if relevant, registered) have been explained.

## ETHICS STATEMENT

This research was reviewed and approved by Al‐basar Institutional Review Board (B‐IRB‐20‐MR‐012). Written ethical permissions were obtained from the hospitals administration. Written informed consent was obtained from the parents for publication of this article and any accompanying images. A copy of the written consent will be available for review by the Editor‐in‐Chief of this journal.

## Data Availability

The datasets used and/or analyzed during the current study are available from the corresponding author on reasonable request.
